# Supplementary material for: Intensive grassland management disrupts below-ground multi-trophic resource transfer in response to drought
Source: Nat Commun. 2022 Nov 16;13:6991. doi: 10.1038/s41467-022-34449-5 (PMC9668848; doi:10.1038/s41467-022-34449-5)
Supplement: Supplementary file 1 — Supplementary material [file 41467_2022_34449_MOESM1_ESM.pdf]

SUPLEMENTARY INFORMATION

Intensive grassland management disrupts below-ground multi-trophic resource transfer in response to drought

Mathilde Chomel*, Jocelyn M. Lavallee, Nil Alvarez-Segura, Elizabeth M. Baggs, Tancredi Caruso, Francisco de Castro, Mark C. Emmerson, Matthew Magilton, Jennifer M. Rhymes, Franciska T. de Vries, David Johnson, and Richard D. Bardgett

SUPPLEMENTARY FIGURES

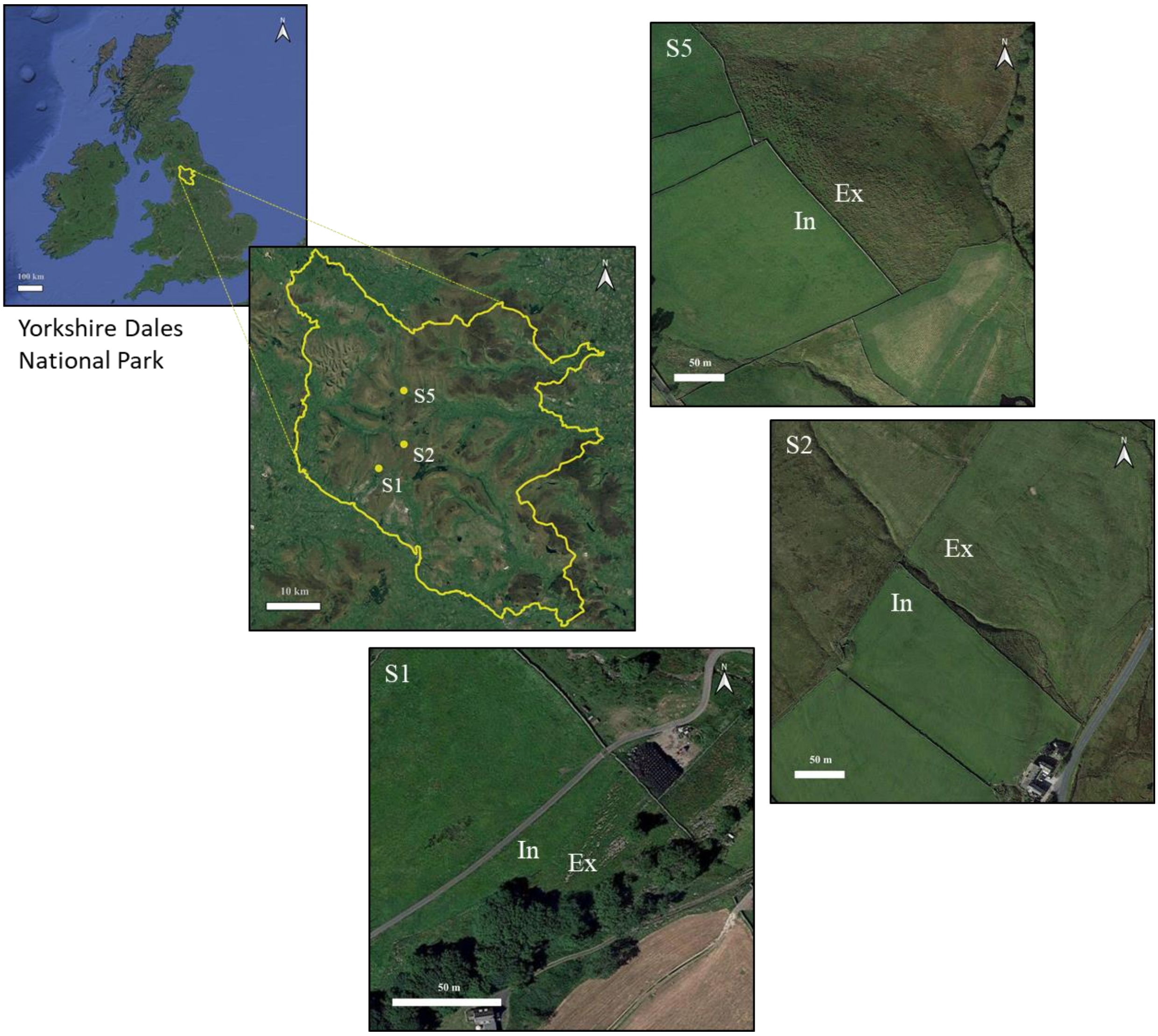

Supplementary Figure 1. Site location.
Map of experimental sites (S1, S2 and S5) and aerial views of the three sites consisting of paired intensively (In) and extensively (Ex) managed grasslands in the Yorkshire Dales National Park, United Kingdom. The maps have been created with the QGIS sotware (www.qgis.org), the Yorkshire Dales National Park outline has been downloaded from the Natural England Open Data Publication (https://naturalengland-defra.opendata.arcgis.com/).

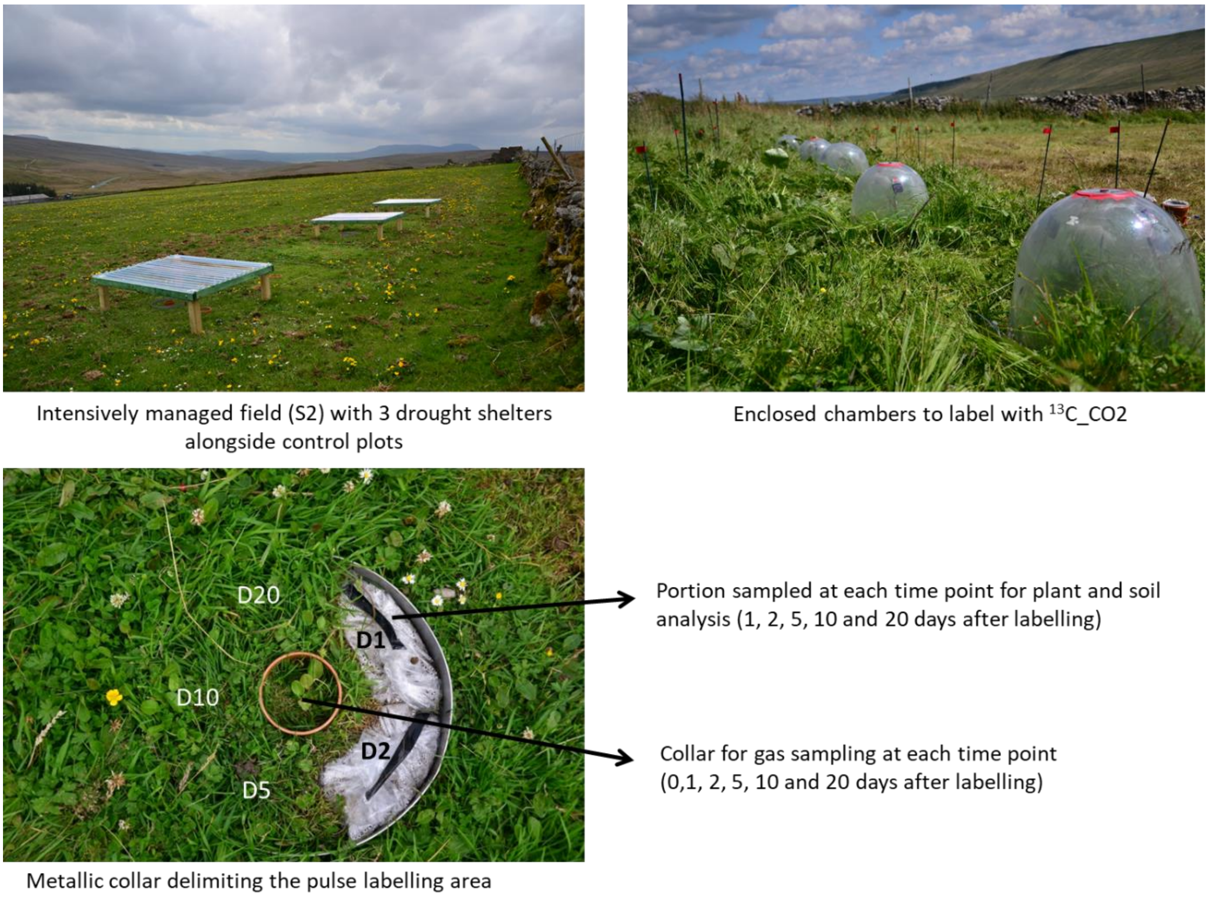

Supplementary Fig. 2. **Experimental plot set-up.**
Pictures of the experimental set-up, $^{13}C-CO_2$ labelling chambers and harvesting plan within the 40cm diameter labelling collar. Pictures from M. Chomel

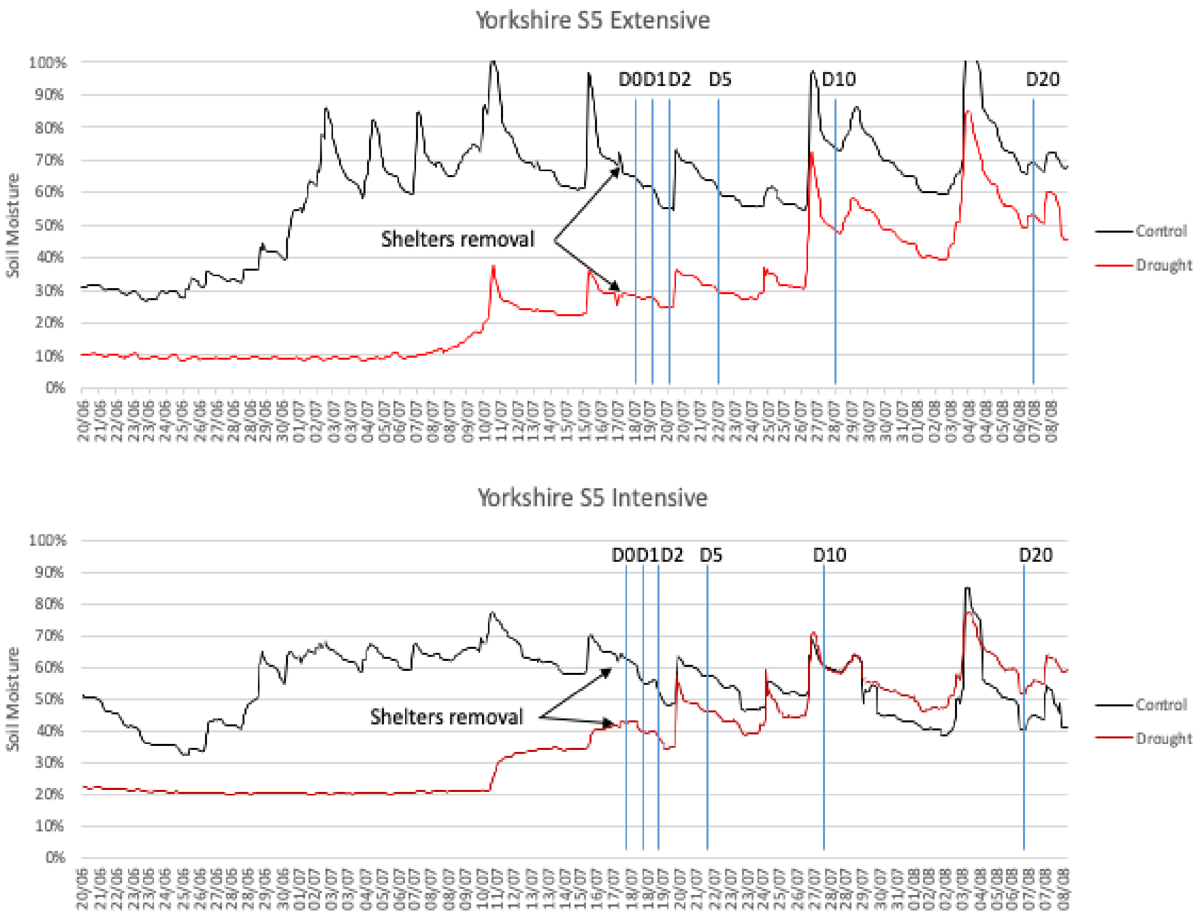

Supplementary Fig. 3. Continuous soil moisture.
Soil moisture of control and droughted plots in site 5 from the second half of the drought until the last sampling which occurred 20 days after the pulse labelling. Sampling dates (D0-D20) are marked with a blue line.

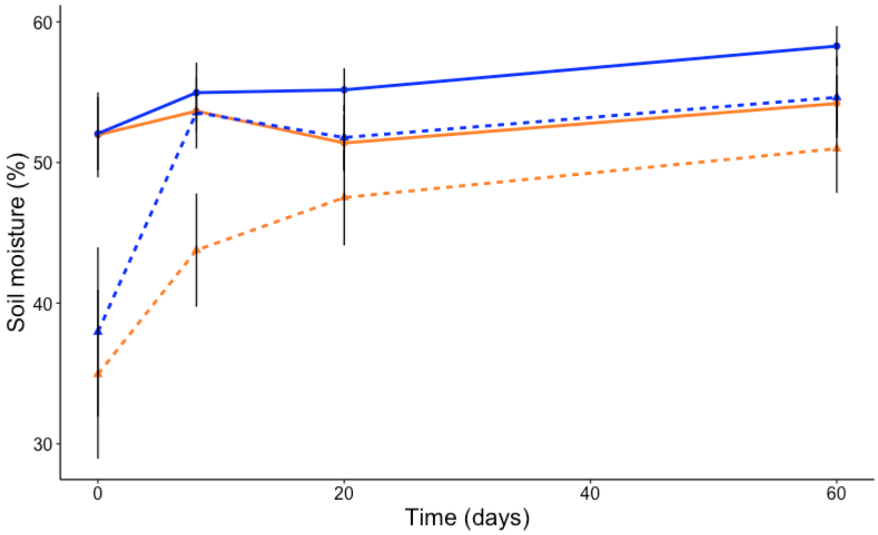

Supplementary Fig. 4. Sequential soil moisture.

Soil moisture (% vol.) after removal of the drought shelters as a function of grassland management (extensive in orange, intensive in blue) and drought (control in solid lines and drought in dotted lines). Lines represent mean of all three sites ± SEM at 4 sampling dates (n = 144 individual samples). The difference between management and treatment was tested using two-sided linear mixed-model followed by multiple comparison Tukey test at each time point. Overall intensively managed grassland generally had higher soil moisture than extensively managed grassland (management: $F_{1,126} = 10.8$, $P = 0.0013$), and drought decreased soil moisture (treatment: $F_{1,126} = 39$, $P < 0.0001$) to the same level in both management regimes (treatment * management: $F_{1,126} = 1.58$, $P = 0.21$). However, the legacy effect of drought on soil moisture persisted longer in extensively compared to intensively managed grassland (Tukey C – D at day 8: P = 0.0046 and 0.63, respectively).

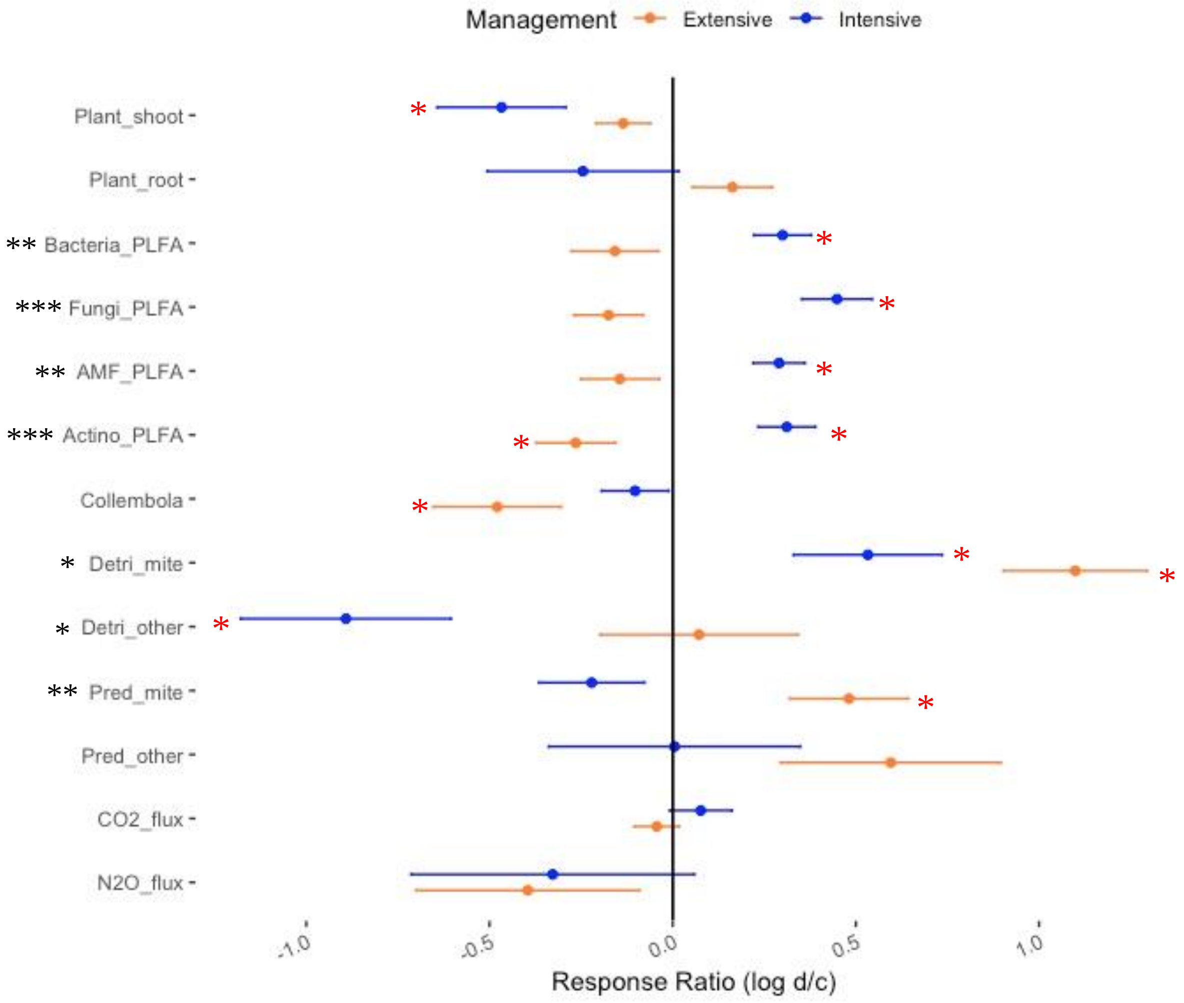

Supplementary Fig. 5. Drought effect on the biomass of plant and soil organisms.
Response ratio of the post-drought effect on the biomass of plants (shoots and roots), soil organisms (PLFA based for microorganisms and trophic group based for mesofauna), and soil CO_2 and N_2O fluxes as a function of grassland management (log (drought/control)). The sign (positive or negative) of the logRR corresponds to the direction of the drought effect on biomasses or fluxes, while a response ratio of zero indicates no post-drought effect. This figure only highlights the strongest effects as logRR values have been calculated on averaged biomass across all sampling dates. Dots represent mean ± SEM (n = 18). Significance for management effect on the logRR from two sided linear mixed-models are reported with *** P < 0.01, **P < 0.01, *P < 0.05. Red asterisks indicate significance for drought effect by the examination of the confidence intervals of predicted means from the linear mixed-effects models, see supplementary table 4 for full statistical outputs. Amf_PLFA = AM fungal PLFA; actino_PLFA = actinobacteria PLFA, detri_mites = decomposer mites; detri_other = other decomposers; pred_mite = predatory mites; pred_other = other predators.

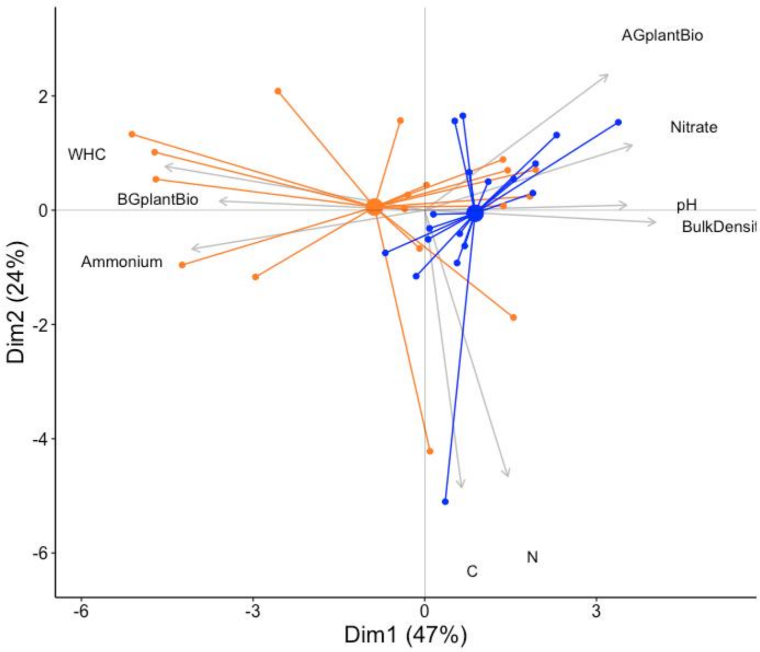

Supplementary Fig. 6. Management effect on soil properties.
Principal component analysis (PCA) from the soil properties and plant communities (extensive in orange, intensive in blue) from the control plots at the first sampling date (n = 36). AGplantBio = Above-ground plant biomass, BGplantBio = below-ground plant biomass, WHC = water holding capacity, C and N = total soil C and N concentrations, and ammonium and nitrate = extractable soil ammonium and nitrate concentrations.

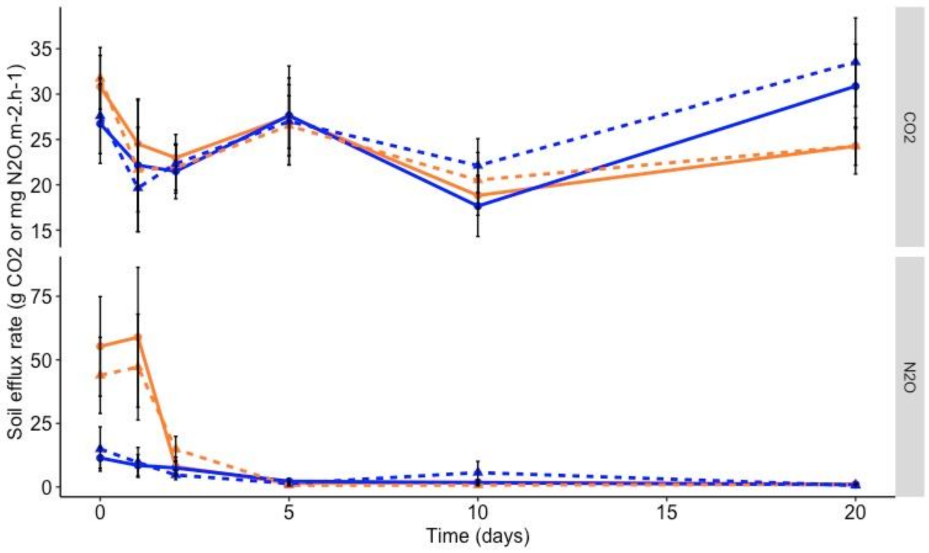

Supplementary Fig. 7. Management effect on soil gas efflux.
Soil CO_2 (upper panel) and N_2O (lower panel) efflux in response to land management (extensive in orange, intensive in blue) and drought (control in plain lines and drought in dotted lines). Lines represent mean ± SEM (n = 216 individual samples). The difference between management and treatment was tested using two-sided mixed model followed by multiple comparison Tukey test at each time point. The effect of land management on soil CO_2 efflux differed on different sampling dates (management * time: $F_{5,190} = 2.86$, P = 0.0162), but there was no significant effect of land management in N_2O efflux at any time (management * time: $F_{5,190} = 1.9$, P = 0.09). N_2O efflux decreased over time (time: $F_{5,190} = 16.7$, P < 0.0001).

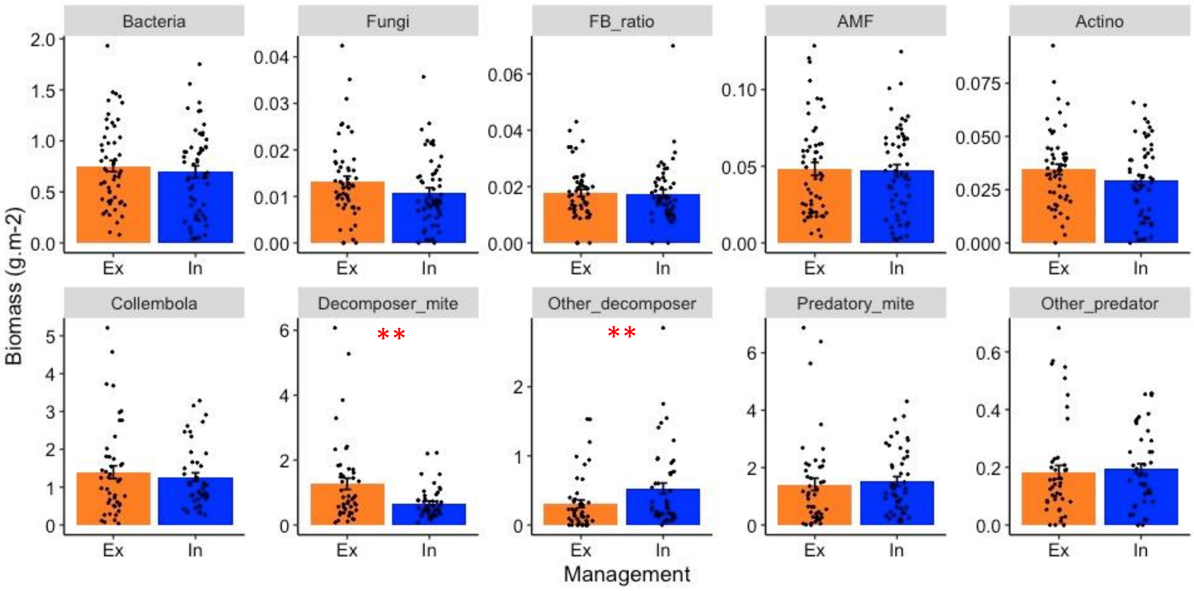

Supplementary Fig. 8. Management effect on soil food web biomass.

Biomass of soil organisms in response to land management (extensive in orange, intensive in blue). Microbial community responses assessed by PLFA are in the upper panel, whereas responses of soil mesofauna are in the lower panel. Bars represent mean ± SEM, dots represent individual observations. Note that values are from control plots only and averaged over replicates and the 6 time points (n = 108 individual samples). Results of the two-sided mixed models are reported with *** P < 0.01, **P < 0.01, *P < 0.05. Intensive management decreased the biomass of decomposer mites (management: $F_{1,82} = 7.03$, 0.0096) and increased the biomass of other decomposer (management: $F_{1,82} = 9.08.7$, P = 0.0034) compared to extensive grassland management.

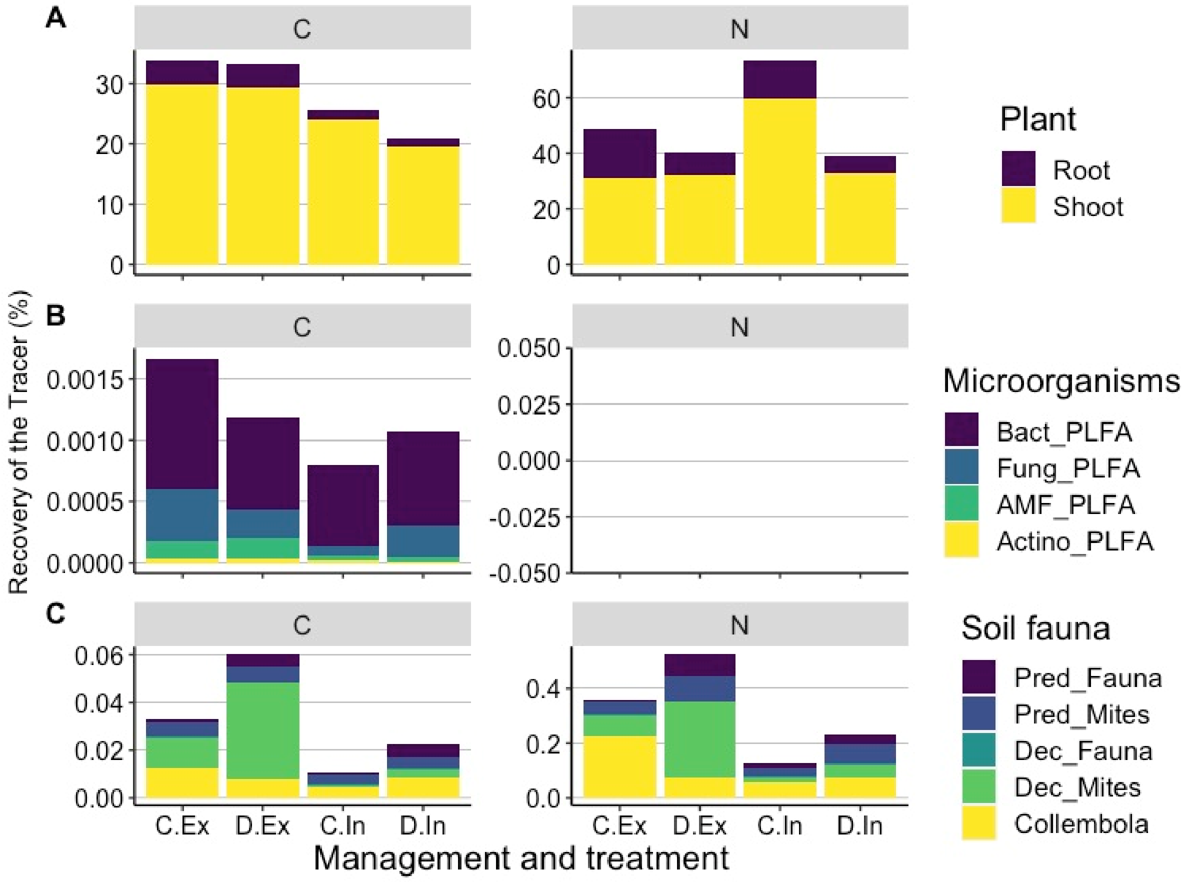

Supplementary Fig. 9. Recovery of C and N tracers in the plant soil system.
Allocation of the ^{13}C and ^{15}N tracer in plants (A), microbial PLFA biomarkers (B), and soil fauna (C) as a function of grassland management (Ex: extensive and In: intensive) and drought (C: control and D: drought). The C and N allocation are presented at the relevant time points for each group: at day 1 for the plant and microorganisms and at day 5 for the soil fauna. Note that the recovery of ^{13}C in microorganisms is expressed in their PLFA, not the total biomass. The ^{15}N enrichment of microbial communities could not be measured. Bars represent mean (n = 180 individual samples). See supplementary Table 4 for detailed statistical results. Dec_mites = decomposer mites; dec_fauna = other decomposers; pred_mite = predatory mites; pred_fauna = other predators; bact_PLFA= bacterial PLFA; fung_PLFA = fungal PLFA; amf_PLFA = AM fungal PLFA; actino_PLFA = actinobacteria PLFA

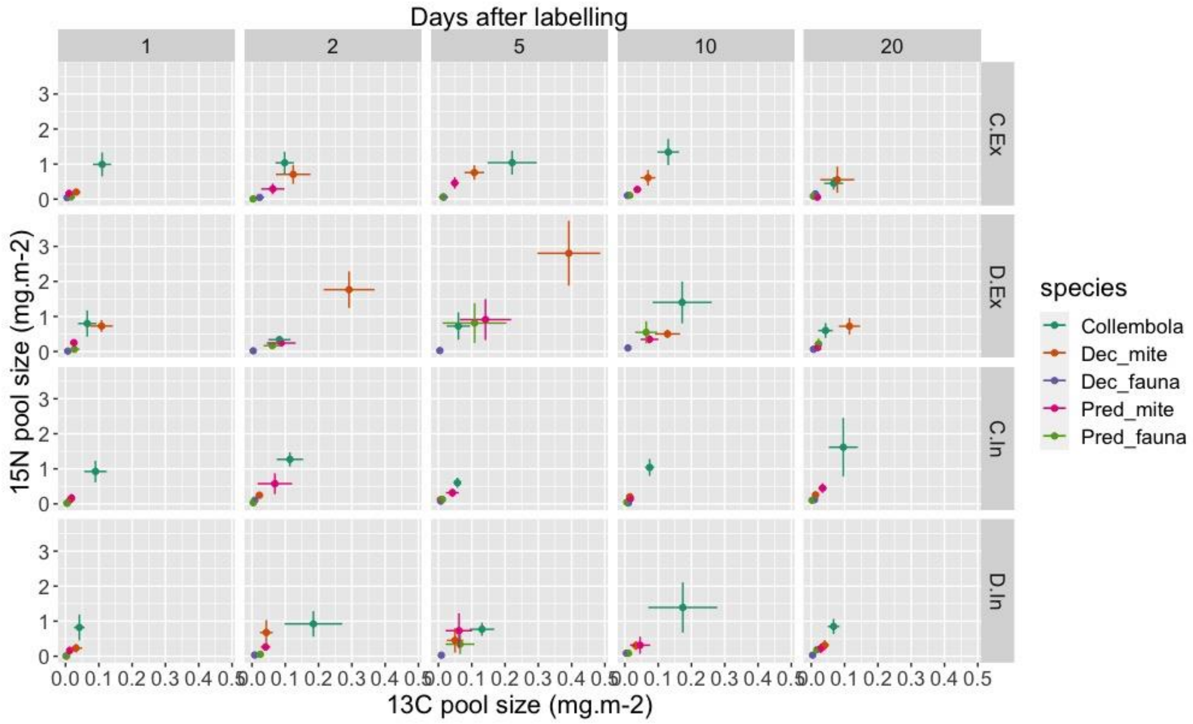

Supplementary Fig. 10. Grassland management and drought effect on tracer pool sizes in soil fauna.

^{13}C and ^{15}N pool size in soil fauna in grassland under intensive (In) or Extensive (Ex) management and after a drought perturbation (D) or under control conditions (C) over time after pulse labelling (days). Points represent mean ± SEM (n = 180 individual samples). See supplementary Table 5 for detailed statistical results. Dec_mites = decomposer mites; dec_fauna = other decomposers; pred_mite = predatory mites; pred_fauna = other predators

SUPPLEMENTARY TABLES

Supplementary Table 1. Management impact on drought response ratio.

Statistical results of the mixed model for the Fig. 2 and supplementary Fig. 5. Grassland management was tested as fixed effect and plot nested in sites as random effect on the response ratio of the drought perturbation on the ^{13}C and ^{15}N enrichment of the different C and N pools in extensively or intensively managed grassland (Fig. 2) and the biomass or gas fluxes (supplementary Fig. 8). Note that logRR values have been calculated on averaged ^{13}C or ^{15}N enrichment or biomass across all sampling dates, hence n = 18.

	Mixed models				Prediction from mixed models					
					Extensive			Intensive		
	Variables	df	F	P	predicted	conf.low	conf.high	predicted	conf.low	conf.high
^{13}C enrichment	Plant shoot	8	12.15	**0.0082**	0.10	-0.14	0.34	**0.46**	**0.22**	**0.70**
	Plant roots	8	10.14	**0.013**	0.10	-0.44	0.65	**-0.66**	**-1.21**	**-0.11**
	Bacteria	8	24.40	**0.001**	-0.03	-0.46	0.39	**-0.58**	**-1.01**	**-0.16**
	Fungi	8	0.06	0.82	-0.26	-0.83	0.31	-0.33	-0.90	0.24
	AM fungi	8	2.78	0.13	0.05	-0.76	0.86	-0.58	-1.39	0.23
	Actino	8	7.03	**0.038**	0.19	-0.53	0.90	**-0.92**	**-1.73**	**-0.10**
	Collembola	8	0.47	0.51	**-0.44**	**-0.87**	**-0.02**	**-0.62**	**-1.05**	**-0.20**
	Detritivorous mites	8	0.13	0.73	0.08	-0.55	0.72	-0.08	-0.71	0.56
	Other detritivores	8	0.25	0.63	-0.42	-1.45	0.62	-0.15	-1.13	0.82
	Predatory mites	8	2.46	0.16	0.04	-0.54	0.61	-0.41	-0.98	0.17
	Other predators	8	1.45	0.26	0.38	-0.50	1.25	-0.32	-1.19	0.56
	CO_2 flux	8	5.53	**0.047**	0.18	-0.13	0.49	**0.44**	**0.13**	**0.75**
^{15}N enrichment	Plant shoot	8	0.12	0.74	-0.04	-0.21	0.13	-0.08	-0.25	0.09
	Plant roots	8	0.21	0.66	-0.33	-0.84	0.18	-0.17	-0.68	0.34
	Collembola	8	0.01	0.91	-0.27	-0.63	0.08	-0.30	-0.66	0.05
	Detritivorous mites	8	0.12	0.74	-0.21	-0.61	0.19	-0.11	-0.51	0.29
	Other detritivores	8	1.70	0.23	-1.11	-2.63	0.42	0.12	-1.41	1.64

	Predatory mites	8	1.02	0.34	0.04	-0.53	0.61	-0.33	-0.90	0.24
	Other predators	8	0.84	0.39	0.08	-0.95	1.12	-0.43	-1.46	0.61
	N_2O flux	8	1.64	0.24	**-0.97**	**-1.67**	**-0.28**	-0.33	-1.03	0.37
Biomass	Plant shoot	8	4.02	0.08	-0.13	-0.40	0.13	**-0.47**	**-0.73**	**-0.20**
	Plant roots	8	2.21	0.18	0.16	-0.23	0.56	-0.24	-0.64	0.15
	Bacteria	8	17.11	**0.003**	-0.16	-0.35	0.04	**0.30**	**0.10**	**0.50**
	Fungi	8	33.80	**<0.001**	-0.17	-0.36	0.01	**0.45**	**0.26**	**0.64**
	AM fungi	8	22.04	**0.002**	-0.14	-0.32	0.03	**0.29**	**0.11**	**0.47**
	Actino	8	34.46	**<0.001**	**-0.26**	**-0.45**	**-0.08**	**0.31**	**0.13**	**0.50**
	Collembola	8	3.74	0.09	**-0.48**	**-0.75**	**-0.21**	-0.10	-0.38	0.17
	Detritivorous mites	8	6.33	**0.03**	**1.10**	**0.71**	**1.49**	**0.53**	**0.14**	**0.92**
	Other detritivores	8	7.90	**0.02**	0.07	-0.47	0.62	**-0.89**	**-1.44**	**-0.35**
	Predatory mites	8	21.90	**0.002**	**0.48**	**0.18**	**0.78**	-0.22	-0.52	0.08
	Other predators	8	2.75	0.14	0.60	-0.04	1.23	0.00	-0.63	0.64
	CO_2 flux	8	1.35	0.28	-0.04	-0.19	0.10	0.08	-0.07	0.22
	N_2O flux	8	0.02	0.89	-0.40	-1.08	0.29	-0.33	-1.01	0.36

Supplementary Table 2. PCoA variables significance.
Statistical results of the monte carlo test of the soil fauna and microbial groups assessed by PLFA against the two PCoA dimensions (PCoA 1 and PCoA 2) presented in Fig. 3.

Variables	PCoA 1	PCoA 2	r^2	P
Ento_ag	-0.38312	-0.9237	0.2248	0.001 ***
Ento_bg	0.97733	0.21174	0.1486	0.003 **
Pod	-0.95376	-0.30056	0.0671	0.044 *
Sym	0.21319	-0.97701	0.0795	0.035 *
Neel	-0.25827	-0.96607	0.0855	0.018 *
Decomp_mites	-0.9441	0.32966	0.1118	0.008 **
Fungal_mites	0.10846	0.9941	0.2913	0.001 ***
Pred_oribatid	-0.25141	0.96788	0.2035	0.001 ***
Mesostigmatid	-0.79439	0.6074	0.4932	0.001 ***
Arachnid	-0.55808	-0.82979	0.0239	0.368
Pred_col_ad	-0.99987	-0.01634	0.0338	0.225
Pred_col_larv	-0.90724	-0.42062	0.0047	0.81
Detrit_col_ad	-0.89807	-0.43986	0.0427	0.165
Detrit_col_larv	-0.77072	-0.63718	0.0397	0.151
Diptera_larv	-0.98488	-0.17324	0.1031	0.013 *
Bactplfa	0.91175	-0.41075	0.0137	0.536
Fungplfa	0.93964	-0.34215	0.0312	0.245
Amfplfa	0.68143	-0.73189	0.034	0.23
Actinoplfa	-0.22471	0.97442	0.0017	0.932

Ento_ag = epigeic entomobryomorpha; ento_bg = eudaphic entomorbyomorpha; pod = poduromorpha; sym= symphypleona; neel = neelipleona; decomp_mites = decomposer mites; fungal_mites = fungivorous mites; pred_oribatids = predatory oribatids; pred_col_ad = predatory coleoptera; pred_col_larv = predatory coleoptera larvae; detrit_col_ad = detritivorous coleoptera; detrit_col_larv = detritivorous coleoptera larvae; diptera_larv = diptera larvae; bactplfa= bacterial PLFA; fungplfa = fungal PLFA; amfplfa=AM fungal PLFA; actinoplfa = actinobacteria PLFA

Supplementary Table 3. Management impact on ^{13}C and ^{15}N enrichment.
Statistical results of the mixed model of the effect of time and management on the ^{13}C relative enrichment and ^{15}N enrichment of the different carbon and nitrogen pools (See Fig. 4 and 5)

	Variables	Management		Time		Mgmt*Time	
		F	*P*	F	*P*	F	*P*
^{13}C	Plant shoot	9.23	**0.003**	31.4	**<0.0001**	0.01	0.93
	CO_2	111.4	**<0.0001**	110.4	**<0.0001**	1.8	0.14
	Plant roots	0.91	0.34	0.28	0.89	1.54	0.2
	Bacteria	0.83	0.36	0.85	0.49	0.16	0.96
	Fungi	0.14	0.71	9.95	**<0.0001**	0.32	0.9
	AM fungi	7.23	**0.008**	1.87	0.11	0.98	0.44
	Collembola	0.33	0.57	1.26	0.3	0.52	0.72
	Detritivorous mites	26.13	**<0.0001**	3.82	**0.007**	0.55	0.7
	Other detritivores	0.002	0.96	0.65	0.63	0.36	0.84
	Predatory mites	1.71	0.2	6.44	**0.0002**	0.45	0.77
	Other predators	0.13	0.087	2.41	0.059	0.32	0.87
^{15}N	Plant shoot	0.13	0.72	13.1	**<0.001**	0.12	0.98
	N_2O	0.81	0.37	45.9	**<0.001**	0.06	0.81
	Plant roots	0.18	0.68	0.38	0.82	1.06	0.38
	Collembola	0.04	0.84	5.7	**0.02**	0.15	0.7
	Detritivorous mites	2.47	0.12	4.53	**0.036**	0.5	0.036
	Other detritivores	0.86	0.36	17.03	**<0.001**	1.44	0.23
	Predatory mites	1.14	0.29	21.2	**<0.001**	0.2	0.65
	Other predators	0.32	0.57	35.86	**<0.001**	4.33	**0.04**

Supplementary Table 4. Management and drought impact on ^{13}C and ^{15}N recovery.

Statistical results of the mixed model for the supplementary Fig. 9. Treatment, management and their interaction were tested as fixed effects and sites as random effect on the recovery of the ^{13}C and ^{15}N tracer in the different carbon and nitrogen pools.

	Variables	Treatment		Management		Trt * Mgmt	
		F	*P*	F	*P*	F	*P*
C recovery	Plant shoots	0.26	0.62	3.23	0.08	0.57	0.46
	Plant roots	0.07	0.79	8.36	**0.007**	0.53	0.47
	Bacteria	0.09	0.77	0.17	0.69	0.52	0.47
	Fungi	0.42	0.52	1.65	0.21	0.10	0.76
	AM fungi	0.31	0.58	9.07	**0.01**	0.29	0.59
	Actino	1.02	0.32	3.42	0.07	0.16	0.69
	Collembola	0.42	0.52	0.22	0.64	3.87	0.06
	Detritivorous mites	2.28	0.14	21.70	**<0.001**	0.02	0.88
	Detritivorous fauna	0.16	0.69	0.77	0.39	0.40	0.53
	Predatory mites	0.15	0.70	7.20	**0.01**	0.20	0.66
	predatory fauna	0.46	0.50	0.88	0.36	0.15	0.70
N recovery	Plant shoots	4.04	0.05	4.42	**0.04**	3.55	0.07
	Plant roots	7.66	**0.01**	2.63	0.12	0.03	0.85
	Collembola	0.79	0.38	0.30	0.59	2.63	0.12
	Detritivorous mites	1.72	0.20	19.54	**<0.001**	0.61	0.44
	Detritivorous fauna	0.03	0.87	2.99	0.09	0.33	0.57
	Predatory mites	0.97	0.33	2.74	0.11	0.17	0.69
	predatory fauna	0.92	0.35	0.48	0.49	2.66	0.11

Supplementary Table 5. Management and drought impact on ^{13}C and ^{15}N pool size.
Statistical results of the two-sided mixed models for data presented in supplementary Fig. 10. Treatment, management, time and their interaction were tested as fixed effect and sites as random effects on the ^{13}C and ^{15}N pool size of the different fauna groups. Note that the interactions management * time and treatment * time were not significant so they have been removed from the models.

Pool size		df	Treatment		Management		Time		Trt * Mgmt	
			F	*P*	F	*P*	F	*P*	F	*P*
Collembola	^{13}C	169	2.44	0.12	0.01	0.92	2.10	0.08	3.90	**0.05**
	^{15}N	168	5.34	**0.02**	1.69	0.20	2.02	0.09	0.76	0.39
Detritivorous mites	^{13}C	169	17.14	**0.0001**	64.09	**<0.0001**	3.37	**0.01**	1.23	0.27
	^{15}N	170	4.37	**0.04**	30.90	**<0.0001**	2.90	**0.02**	2.40	0.12
Detritivorous fauna	^{13}C	149	1.12	0.29	0.12	0.73	0.97	0.43	0.11	0.74
	^{15}N	148	2.10	0.15	0.97	0.33	2.42	0.05	0.00	0.99
Predatory mites	^{13}C	169	3.02	0.08	4.65	**0.03**	8.49	**<0.0001**	3.08	0.08
	^{15}N	169	0.82	0.37	0.15	0.70	6.32	**0.0001**	5.99	**0.02**
predatory fauna	^{13}C	157	13.03	**0.0004**	6.65	**0.01**	1.69	0.15	0.55	0.46
	^{15}N	158	3.96	**0.05**	1.17	0.28	3.61	**0.01**	1.83	0.18

Supplementary Table 6. Characteristics of the sites. Location and properties of the three paired sites of the study with grassland management contrast.

Site	Management	Coordinates	Pulse labelling date	Soil type	Soil pH	Bulk density	Altitude (m)
1	Intensive	N 54°12.757' W 2°23.459'	19/07/2016	Humose loamy	5.33	0.60	327
1	Extensive	N 54°12.761' W 2°23.444'	19/07/2016	Humose loamy	5.02	0.68	327
2	Intensive	N 54° 15.190' W 2°19.134'	18/07/2016	Humose loamy	5.49	0.34	496
2	Extensive	N 54°15.219' W 2°19.093'	18/07/2016	Humose loamy	5.51	0.31	496
5	Intensive	N 54°20.385' W 2°19.252'	17/07/2016	Humose loamy	4.70	0.52	339
5	Extensive	N 54°20.389' W 2°19.242'	17/07/2016	Humose loamy	4.16	0.18	339
