# Supplementary material for: Intensive grassland management disrupts below-ground multi-trophic resource transfer in response to drought
Source: Nat Commun. 2022 Nov 16;13:6991. doi: 10.1038/s41467-022-34449-5 (PMC9668848; doi:10.1038/s41467-022-34449-5)
Supplement: Supplementary file 3 — Reporting Summary [file 41467_2022_34449_MOESM3_ESM.pdf]

nature portfolio

Corresponding author(s): Chomel Mathilde

Last updated by author(s): 19/10/22

Reporting Summary

Nature Portfolio wishes to improve the reproducibility of the work that we publish. This form provides structure for consistency and transparency in reporting. For further information on Nature Portfolio policies, see our Editorial Policies and the Editorial Policy Checklist.

Statistics

For all statistical analyses, confirm that the following items are present in the figure legend, table legend, main text, or Methods section.

n/a	Confirmed	
☐	☒	The exact sample size (n) for each experimental group/condition, given as a discrete number and unit of measurement
☐	☒	A statement on whether measurements were taken from distinct samples or whether the same sample was measured repeatedly
☐	☒	The statistical test(s) used AND whether they are one- or two-sided<br>*Only common tests should be described solely by name; describe more complex techniques in the Methods section.*
☐	☒	A description of all covariates tested
☐	☒	A description of any assumptions or corrections, such as tests of normality and adjustment for multiple comparisons
☐	☒	A full description of the statistical parameters including central tendency (e.g. means) or other basic estimates (e.g. regression coefficient) AND variation (e.g. standard deviation) or associated estimates of uncertainty (e.g. confidence intervals)
☐	☒	For null hypothesis testing, the test statistic (e.g. F, t, r) with confidence intervals, effect sizes, degrees of freedom and P value noted<br>*Give P values as exact values whenever suitable.*
☒	☐	For Bayesian analysis, information on the choice of priors and Markov chain Monte Carlo settings
☒	☐	For hierarchical and complex designs, identification of the appropriate level for tests and full reporting of outcomes
☒	☐	Estimates of effect sizes (e.g. Cohen's d, Pearson's r), indicating how they were calculated

Our web collection on statistics for biologists contains articles on many of the points above.

Software and code

Policy information about availability of computer code

Data collection	No software was used for data collection
Data analysis	All analyses were performed using R software (version 4.1.0. R core Team, 2017)

For manuscripts utilizing custom algorithms or software that are central to the research but not yet described in published literature, software must be made available to editors and reviewers. We strongly encourage code deposition in a community repository (e.g. GitHub). See the Nature Portfolio guidelines for submitting code & software for further information.

Data

Policy information about availability of data

All manuscripts must include a data availability statement. This statement should provide the following information, where applicable:

- Accession codes, unique identifiers, or web links for publicly available datasets
- A description of any restrictions on data availability
- For clinical datasets or third party data, please ensure that the statement adheres to our policy

The datasets generated during and/or analysed during the current study are available from the corresponding author on reasonable request

Field-specific reporting

Ecological, evolutionary & environmental sciences study design

All studies must disclose on these points even when the disclosure is negative.

Study description	We quantified how historical grassland management modifies transfers of recent photosynthate and soil nitrogen through plants and the soil food web in response to drought, using in situ 13C and 15N pulse-labelling in paired intensively and extensively managed fields. For this we chose 3 geographically distinct grasslands in the Yorkshire Dales, northern England. At each site there was an extensively managed field immediately adjacent to an intensively managed field. In each field we installed 3 transparent roofs (1.5m*1.3m) to intercept all the rainfall, alongside delimited control plots. In the central part of each plots, a 40 cm diameter collar was inserted at the beginning of the experiment to delimit the pulse labelling area. The rain shelters were in place for 60 days. After the release of the drought, a solution of NH4 15N-NO3 was injected in the soil in each plot. Plants were exposed to 13C_CO2 for 2-3 hours, by injection of the isotope into a closed chamber placed on the top of the collar. Immediately after the pulse labelling and after 1, 2, 5, 10 and 20 days, samples of plant shoots and roots, soil CO2 efflux, bulk soil, and soil fauna were taken from each plot to trace the 13C and the 15N within the plant-soil system.<br>In total there were 36 plots (3 sites * 2 managements * 2 treatments * 3 replicates) and 6 (for plant shoot and gas sampling) or 5 (for all the other data) sampling dates
Research sample	Samples have been collected from a controlled field experiment in extensively and intensively managed grasslands. These representative grasslands from the UK have been chosen on the base of low or high inputs of fertiliser/ grazing pressure respectively following previous study showing changes in food web structure with grassland managment intensity (de Vries et al 2006; de Vries et al, 2012; Ward et al 2016). From these plots, several samples have been taken after the pulse labelling to trace the stable isotope throught the plant-soil system:<br>- Plant shoot and roots samples to represent the whole plant community<br>- Gas samples to represent gas exchange between plant, soil and atmosphere during 30min<br>- Soil samples to extract soil mesofauna and PLFA for microbial communities. The focus has been made on mesofauna, mainly Collembola and mites, two main groups in term of abundance of soil fauna, and microbial community to be representative of the soil community in these systems.<br>-Soil samples that has been sieved, homogeneised and used for other analyses to be representative of soil chemical and physical properties
Sampling strategy	We choose 3 geographically distinct managed grassland sites in the Yorkshire Dales, northern England (more than 5 km apart, and with a maximum of 15km apart), to encompass the heterogeneity of mesotrophic grasslands within this region. Within each site we choose an area where an extensively managed grassland was adjacent to an intensively grassland to control for differences in soil type and biotic/abiotic conditions, thus ensuring the only difference was in land management. In each field, we had three replicates drought and three replicates control plots to encompass the variability of the soil fauna. Within each 1.5 m * 1.3 m plot, a 40cm diameter collar delimited the pulse labelling and sampling area in intensively and extensively managed grasslands. 40cm diameter has been estimated to be a good compromise between enough space to encompass the variability in plant and soil community and not too much space which could cause dilution of the stable isotope and compromise the stable isotope tracing (based on previous work, de Vries et al, 2012, Morrien et al 2017).<br><br>To quantify the dynamics of the stable isotopes through the plant-soil system, we sampled an intensive time series comprising 6 sampling times: at the end of the pulse-labelling, and after 1, 2, 5, 10, 20 days. This time-course was chosen to ensure we maximised the likelihood of capturing peak isotopic enrichment within all the trophic groups within the soil system, and was based on previous experiments (Chomel et al 2019, DOI: 10.1111/gcb.14754). Plant and soil samples consisted of 1/5th of the 40cm diameter collar (aprox. 300g of soil) which is enough to characterise the plant and fauna communities in these systems based on the literature.
Data collection	Pulse labeling was done during three consecutive days in each site for logistical reasons. Data collection on site was mainly recorded by MC and NAS on notebooks, with the help of JML and JMR. Metadata was recorded on notebooks as well. Gas samples were taken on site and stored until analysis. To minimise loss of stable isotope, and to ensure that what we measured reflected the time of sampling, soil samples were immediately transported to a laboratory to extract the fauna and soil sieved for KCl extractions. The extracts, plant material, and soil were frozen the same day until their analysis.<br>MC, NAS and HS completed analyses of samples in the laboratory and collected data and metadata on lab notebooks (pH meter, sample numbering, time of analysis etc.), and data from instruments were dowloaded and stored as excel files on a computer as soon as the analyses were performed (GC-MS, Picarro, TOC, CHN, colorimetric segmented flow analyser etc.).
Timing and spatial scale	We choose 3 geographically distinct managed grassland sites in the Yorkshire Dales, northern England (more than 5 km apart, and with a maximum of 15km apart). Exclusion shelters were installed on the 17-18-19th of May 2016 and removed on the 17-18 and 19th of July 2016. The timing was based to simulate a 100-year drought event in these systems (Bloor and Bardgett 2012, de Vries et al 2018, Cole et al, 2019). Drought and control plots were installed in the same area in each field to be enclosed and protected from cattle or sheep. Each control plot was paired with an adjacent drought plot, and the 6 plots in each field were installed in an area from 30 to 50 m-2.<br>15N labelling was applied 5 hours after the release of the drought on the 17-18th and 19th of July at around 6pm, and 13C labelling was applied on the 18th-19th-20th of July at around 10am and all samples (Day0) collected by around 2.30pm. Samples were subsequently taken 1, 2, 5, 10 and 20 days after, at around 1pm, by taking 1/5th of the 40cm diameter collar. The last sampling day (D20) were the 8-9-10th of August 2016. This time-course was chosen to ensure we maximised the likelihood of capturing peak isotopic enrichment within all the trophic groups within the soil system, and was based on previous experiments (Chomel et al 2019, Morrien et al, 2017, Fuchslueger et al, 2014).

Data exclusions	Only 5 outliers were removed from the whole dataset. After noticing an important deviation from the rest of the datapoint, i.e. outside of 3 standard deviation from the mean (99.7%), the data point was the object of a thorough investigation to understand why this could be an outlier. The evidence suggested these 5 outliers were likely due to measurement error and so the data points were removed from the dataset.
Reproducibility	All the instruments used to analyse the samples were checked for accuracy in each run by analyzing standards with known concentration and 13C and 15N signature.
Randomization	This is not relevant to our study as it is a field experiment
Blinding	To insure blinding, all the samples were analyzed randomly

Did the study involve field work? ☒ Yes ☐ No

Field work, collection and transport

Field conditions	The field experiment was carried out across a series of mesotrophic grasslands located in the Yorkshire Dales, northern England, with a mean annual temperature 7.3C and mean annual precipitation 1382 mm. Sites were all humose loamy brown earth (pH ~5.47; 11.4 % total C; 0.76 % total N).
Location	Sites :<br>1 Intensive; N 54°12.757' W 2°23.459' ; Altitude: 327m<br>1 Extensive ;N 54°12.761' W 2°23.444' ; Altitude: 327m<br>2 Intensive; N 54° 15.190' W 2°19.134' ;Altitude: 496m<br>2 Extensive; N 54°15.219' W 2°19.093' ;Altitude: 496m<br>5 Intensive ;N 54°20.385' W 2°19.252' ; Altitude:339m<br>5 Extensive ;N 54°20.389' W 2°19.242' ;Altitude: 339m
Access & import/export	Within the Yorkshire Dales region we first searched for mesotrophic grassland where we could observe an intensively managed grassland next to extensively managed grassland. We then asked the owners of the field to specify the management of each of these field to confirm the difference of management and asked the permission to perform the field experiment on their land. We had their authorisation to take plant and soil samples during the course of the experiment.
Disturbance	The disturbance was only minimal as after the end of the experiment we filled back holes done for soil sampling with soil from the surroundings. Owner of the land were aware of extraction of plant and soil material.

Reporting for specific materials, systems and methods

We require information from authors about some types of materials, experimental systems and methods used in many studies. Here, indicate whether each material, system or method listed is relevant to your study. If you are not sure if a list item applies to your research, read the appropriate section before selecting a response.

Materials & experimental systems

n/a	Involved in the study	
☒	☐	Antibodies
☒	☐	Eukaryotic cell lines
☒	☐	Palaeontology and archaeology
☒	☐	Animals and other organisms
☒	☐	Human research participants
☒	☐	Clinical data
☒	☐	Dual use research of concern

Methods

n/a	Involved in the study	
☒	☐	ChIP-seq
☒	☐	Flow cytometry
☒	☐	MRI-based neuroimaging
